# A tailored, interactive health communication application for patients with type 2 diabetes: study protocol of a randomised controlled trial

**DOI:** 10.1186/1472-6947-13-24

**Published:** 2013-02-13

**Authors:** Nina Weymann, Martin Härter, Jörg Dirmaier

**Affiliations:** 1 Department of Medical Psychology (W 26), University Medical Center Hamburg-Eppendorf, Martinistr. 52, Hamburg, 20246, Germany

**Keywords:** Type 2 diabetes mellitus, Randomized controlled trial, Study protocol, Patient information, Web

## Abstract

**Background:**

Type 2 diabetes is an increasingly common chronic condition whose prognosis can be improved by patient involvement and self-management. Patient involvement can be fostered by web-based Interactive Health Communication Applications (IHCAs) combining health information with decision support, social support and/or behaviour change support. They reach great numbers of patients at low cost and provide high-quality information and support at the time, place and learning speed patients prefer. Still, online tools often suffer from high attrition. Tailoring content and tone of IHCAs to the individual patient´s needs might improve their effectiveness. This study aims to test the effectiveness and usage of a tailored IHCA combining health information with decision support and behaviour change support for patients with type 2 diabetes.

**Methods/design:**

The effectiveness and usage of the tailored IHCA will be tested against a standard website with identical content in a single-blinded randomized trial with a parallel design. The content covers information on type 2 diabetes, its complications and sequelae, and its treatment options including health behaviour. In the intervention group the content is delivered in dialogue form, tailored to relevant patient characteristics (health literacy, attitudes towards self-care, and barriers to insulin treatment). In the control group the different sections are presented in a content tree, without any tailoring. Participants are blinded to group assignment. Eligibility criteria are age ≥ 18 years, self-reported type 2 diabetes, and Internet access. The study aims to include 414 participants in order to detect the expected small effect (Cohen’s d=0.2), with measurements at baseline, directly after the first visit, and at 3-month follow-up. The primary hypothesis is that the tailored IHCA has larger effects on diabetes knowledge and patient empowerment (primary outcomes) than the standard website. Secondary outcomes are website usage as well as decisional conflict and preparation for decision making. All measurements are online self-report questionnaires.

**Discussion:**

IHCAs are a promising way to foster diabetes knowledge and self-management competencies. The present trial tries to increase the knowledge on how to develop more effective IHCAs for patients with type 2 diabetes.

**Trial registration:**

International Clinical Trials Registry DRKS00003322

## Background

Diabetes and its secondary diseases are a leading cause of morbidity and mortality in many countries. The number of people living with diabetes more than doubled during the last three decades [1]. Projections expect the prevalence to rise from 2.8% in 2000 to 4.4% in 2030 [2]. Type 2 diabetes accounts for 90 to 95% of diabetes cases [3]. Patients, practitioners, scientists and politicians have called for more active patient involvement in the making of medical decisions as well as in the management of diabetes. Patient involvement has been shown to reduce fasting blood glucose levels, glycated hemoglobin levels, and the need for diabetes medication [4]. Two main aspects of patient involvement are self-management and shared decision-making (SDM). Both for shared decision-making and for self-management patients need to be informed about their disease, its course, and the treatment options at hand, including their advantages and disadvantages. However, due to limited resources in health care, large numbers of patients still do not have access to feasible diabetes education [5,6].

In times of rapidly growing Internet penetration, the web holds the opportunity to deliver health information and support to large numbers of participants on comparatively low cost and at the time, place and learning speed the individual users prefer. Trials of systematically and thoroughly developed online health interventions show small but consistent effects on clinical outcomes [7-9] even in older populations that are generally thought to be less inclined to use the web [10]. Murray et al. [11] reviewed the effects of a format that combines health information with at least one other type of support, e.g., social support, decision support, or behaviour change support (= “Interactive Health Communication Applications”, IHCAs) [11]. They found that IHCAs can have positive effects on knowledge, social support, clinical, and behavioural outcomes.

Still, the effectiveness of those online applications is limited by high attrition rates [12,13], and few users visit a health intervention website more than once [14,15]. Since the effect of online interventions increases with dose (longer stays, repeated website visits, total contact hours) [15,16], effectiveness is maximized if patients work intensively with the information offered [17,18] and return for repeated visits [19,20]. Individualization and personalization of information as well as an interactive presentation have been found to effectively increase exposure to and effectiveness of interventions [21,22]. These three strategies can be subsumed under the concept of tailoring [23].

### Aims of the trial

This trial tests an IHCA presenting diabetes information, self-management education and decision support in a dialogue-based, tailored format against a website presenting the same information in a content tree without dialogue or tailoring. The primary hypothesis is that the interactive and individualized delivery format has larger effects on diabetes knowledge and patient empowerment than the standard website. Exploratory research questions are if usage is higher for the interactive and individualized delivery format and whether users facing a health decision experience less decisional conflict and feel better prepared for the consultation after using the interactive and individualized site rather than the standard website.

## Methods/Design

### Study design

**Figure 1:**
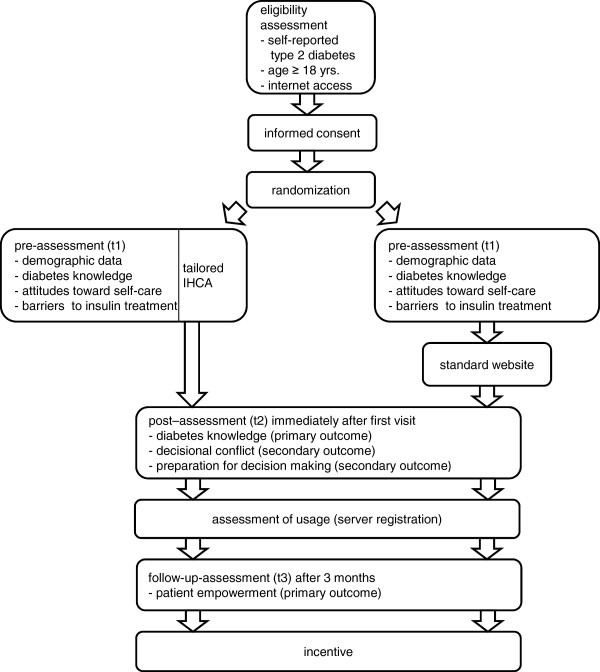
Study procedures.

We chose a single-blinded two-armed randomised controlled trial (RCT) with a parallel design. We aim to include N=414 participants. Measurements are scheduled immediately before the first use of the system, immediately after and at three month follow-up. Diabetes knowledge (primary outcome), decisional conflict, and preparation for decision making (secondary outcomes) are assessed immediately after the first visit. Patient empowerment (secondary outcome) is assessed three months after the first visit (see figure 1).

### Study procedures

After providing an informed consent and completing the pre-assessment (eligibility criteria, demographic data, time since diagnosis, treatment) the participants are randomly assigned to the tailored IHCA or the standard website with the content tree. In the intervention group, the questions used for tailoring are presented during the dialogue. Participants assigned to the control condition where no tailoring takes place fill in these questionnaires immediately before visiting the website in order to control for baseline disparities between intervention and control group. Immediately after their first visit to the IHCA or the standard website all participants are asked to fill in the post-assessment.

All participants receive an e-mail three months after their first visit asking them to fill in the follow-up online questionnaire. Because non-monetary incentives have been shown to reduce attrition in online trials [24,25], participants who have answered all questionnaires receive a 10 € amazon gift voucher. The voucher code is sent to them by e-mail at the end of the study. Figure 1 gives an overview of the study procedures.

Participants are free to use the intervention as often and as long as they wish, also between the post and follow-up assessment. Information on frequency and duration of usage is gathered via server registrations. Usage data, data from the self-assessment questionnaires, and personal data such as name and e-mail address are saved separately. Data are pseudonymesed. After data collection, personal data will be deleted. If a participant withdraws his or her informed consent to study participation his or her data shall be erased immediately. All data will be erased five years after the end of the study. The study was approved by the Hamburg Medical Chamber ethics committee.

### Treatment allocation

The informed consent informs participants that they will be randomly assigned to one of two presentation formats holding the same content. The two formats are not further elucidated so participants do not know whether they are in the intervention or control group. Randomisation is performed by computer.

### Recruitment

In Germany, health care for chronically ill patients is organized in different sectors, mainly in acute-care clinics and rehabilitation centres for in-patient care, and primary care and diabetology practices for out-patient care. Treatment in acute-care clinics, in primary care practices, and in diabetology practices is funded by health insurance while rehabilitation in rehabilitation centres is usually funded by pension funds. Study aim is to include patients from all sectors. Recruitment takes place via support from different health insurance companies, pension funds, primary care practices and diabetology practices, hospitals and rehabilitation centres, and self-help groups. The study is advertised in various media such as newspapers, magazines, patient websites, and flyers. Information on the study is available on the study website http://www.entscheidungshilfe.info.

### Study Population

Eligibility criteria are age ≥ 18 years, access to the Internet, and a self-reported diagnosis of type 2 diabetes. According to the American Diabetes Association [3,26], type 2 diabetes should be diagnosed if a fasting plasma glucose of ≥ 126 mg/dl or a non-fasting plasma glucose of ≥ 200 mg/dl or a plasma glucose of ≥ 200 mg/dl two hours after oral intake of 75g glucose (oral Glucose Tolerance Test, oGTT) is measured. Supposing that most patients cannot give these exact numbers and that self-reported diagnoses are a valid criterion [27], we decided to rely on self-reported diagnosis of type 2 diabetes as inclusion criterion.

### Description of the intervention and control condition

The content of both the tailored IHCA and the standard website covers basic information on diabetes (pathophysiology, epidemiology, subtypes, symptoms) and its sequelae (neuropathy, nephropathy, retinopathy, heart and vessel problems, sexual dysfunction, and depression), information on health behaviour and lifestyle changes, and treatment options (see section Chapters and sections). The look of the website (colours, typing, figures and pictures) is identical in both conditions. After registration, each participant receives a password via e-mail with which he/she can log into the system as often as he/she wishes.

#### Chapters and sections

1. Introduction: What is this website?

1.1. Where does the information on this site come from?

1.1.1. What are treatment guidelines?

1.1.2. What are disease management programmes?

2. Basics

2.1. Different diabetes types

2.1.1. Type 1

2.1.1. Type 2

2.1.1. Other types of diabetes

2.1. How do I know I have type 2 diabetes?

2.1.1. The most important signs

2.1.1. Other signs

2.1. What causes type 2 diabetes?

2.1.1. What causes insulin resistance?

2.1.1. Risk factors

2.1. How many people live with type 2 diabetes?

2.1.1. Diabetes is on the rise

2.1. How is type 2 diabetes diagnosed?

2.1.1. Fasting plasma glucose

2.1.1. Oral glucose tolerance test (oGTT)

2.1.1. Measurement units for blood sugar

2.1.1. HbA1c

2.1.1. Urine analysis

2.1. Diabetes ABCs

2.1.1. „A“ is for HbA1c

2.1.1. “B” is for blood pressure

2.1.1. „C“ is for cholesterol

2.1. Blood sugar control

2.1.1. What is it good for?

2.1.1. How to do it

2.1.2. At the doctors´ practice

2.1.2. At home

3. How is type 2 diabetes treated?

3.1. What are the goals of diabetes treatment?

3.1. What can you do to treat your diabetes?

3.2. How do I keep a healthy diet?

3.2. Why is exercise important?

3.2. Why is smoking so bad if you have diabetes?

3.1. When should you consider taking pills?

3.2. Pills to treat type 2 diabetes

3.2. How much do they lower blood sugar levels?

3.2. Biguanide /metformin

3.2. Sulphonylureas

3.2. Glinides (repaglinide, nateglinide)

3.2. Glitazone

3.2. α-glucosidase inhibitor (AGI)

3.2. Dipeptidyl peptidase-4 inhibitor

3.2. Exenatide, liraglutide

3.1. Insulin treatment

3.2. Human insulin and insulin analogues

3.2. When is insulin treatment initiated?

3.2. Hopes and fears when starting insulin treatment

3.2. How is insulin administered?

3.2. Different types of insulin and their effects

3.2. How does insulin act in the body?

3.2. Insulins with different durations of action

3.2. Insulin treatment and blood sugar control

3.2. Combining pills and insulin

3.1. Summary and overview of the treatment options

4. Acute complications and sequelae

4.1. Which acute complications can occur?

4.1.1. Low blood sugar

4.1.1. High blood sugar

4.1. Which sequelae can occur?

4.1.1. Coronary heart disease and stroke

4.1.1. Neuropathy

4.1.1. Nephropathy

4.1.1. Retinopathy

4.1.1. Diabetic foot

4.1.1. Skin diseases

4.1.1. Sexual health

4.1.1. Depression

5. Additional information and literature

5.1. Associations and self-help

5.1. Web sites

5.1. Journals

5.1. Books

6. Glossary

7. Legal notice

8. References

#### Intervention condition

In the intervention the delivery format is a dialogue-based, tunnelled design tailoring the content and tone of the dialogue to relevant patient characteristics. A tunnelled design where the user is guided through the content was found to increase website use and knowledge gained from a website more than a design with more user control [28]. Still, it might also annoy the user and evoke resistance [29]. Consequently we decided to give the user some control over the path he/she takes through the dialogue. At the end of each text passage the user chooses one of at least three reply options and receives a tailored answer. The answers mirror what the user has said, convey esteem and empathy and build an individualized bridge to the next content block.

**Figure 2:**
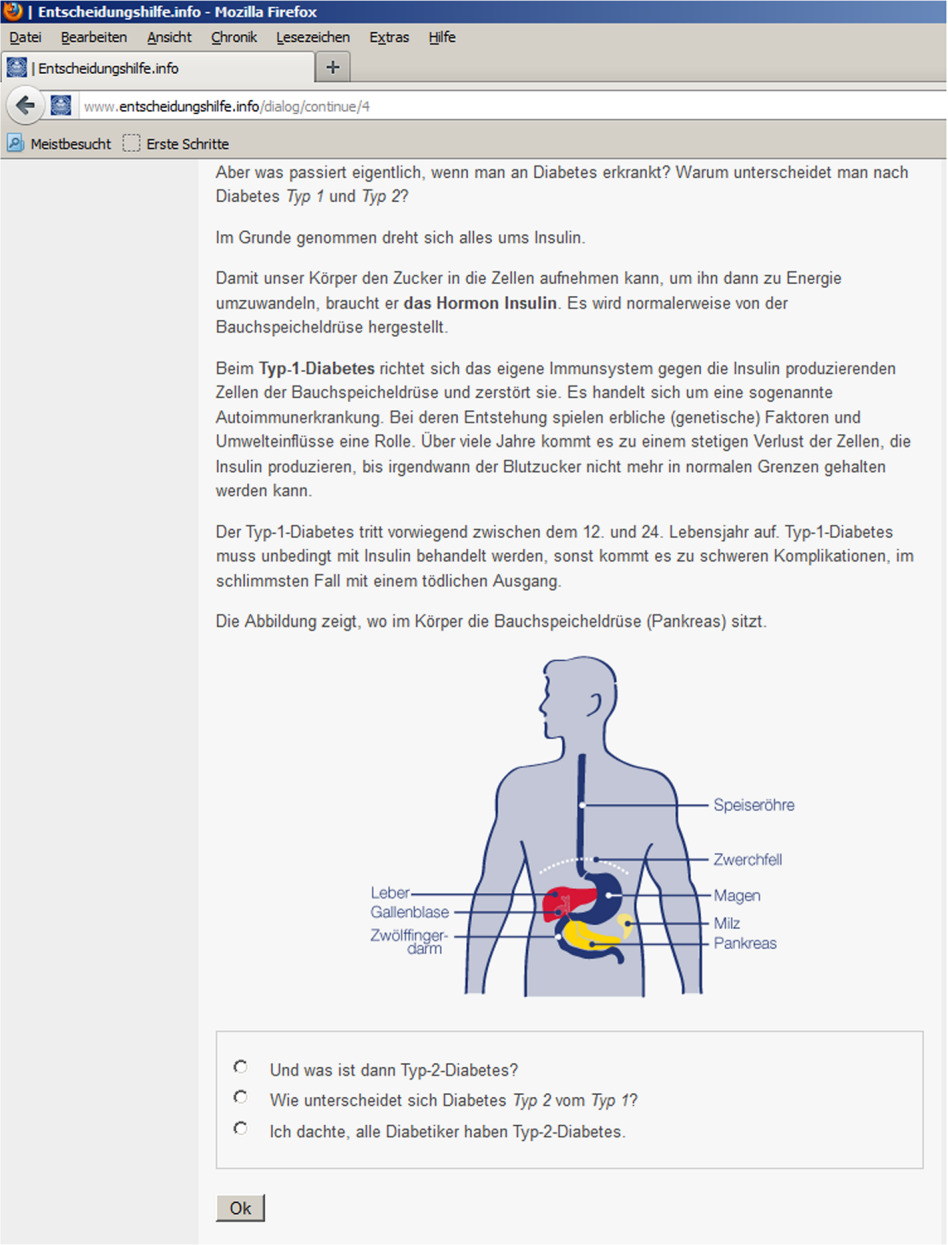
Dialogue window.

*Tailoring* is performed on the following patient characteristics: health literacy, attitudes towards self-care, and, if insulin treatment is a relevant topic, psychological barriers to it. The questionnaires that assess patient characteristics are presented during the dialogue: In the beginning of the respective section (e.g. diabetic foot), the participant is asked about his or her knowledge or attitude toward the topic. The following section is then modified according to his/her answer. Figure 2 shows a dialogue window.

**Table 1:** Example of self-care tailoring

Item	People with diabetes are advised to regularly check their feet and the inside of their shoes. People differ a lot with respect to the importance they attach to “good advice“ of this kind. How important is this advice for you personally?		
Reply options	not important	a little important	important or very important
Tailored answer	OK, so this recommendation is not important for you. Maybe you are very aware of the inconvenience of daily foot care. You are right there; it takes some effort in the beginning. At the same time it helps a lot to prevent diabetic foot syndrome. A diabetic foot can be painful and can lead to amputation. There is a great benefit for the comparatively small effort of taking care of your feet. For many people the first step is the hardest. Once you get used to it, the effort does not seem so great anymore.	OK, so this recommendation is a little important for you. Maybe you are aware of the inconvenience of daily foot care. At the same time it helps a lot to prevent diabetic foot syndrome. A diabetic foot can be painful and can lead to amputation. There is a great benefit for the comparatively small effort of taking care of your feet. For many people the first step is the hardest. Once you get used to it, the effort does not seem so great anymore.	You are right, this recommendation is really important. Looking after your feet can be inconvenient but helps a lot to prevent diabetic foot syndrome. A diabetic foot can be painful and can lead to amputation. There is a great benefit for the comparatively small effort of taking care of your feet.

The user´s attitudes towards self-care are assessed with items that we adapted from the Summary of Diabetes Self-care Activities Measure (SDSCA) [30] to match the respective content section (see Table 1). The question is always how important a certain self-care activity or advice is for the individual user. Every item has three reply options: “important or very important”, “a little important”, and “not important”. The goal and techniques are inspired by Motivational Interviewing [31]. Motivational interviewing is a counseling method for addressing ambivalence about change.

For example, if a user attaches great importance to the self-care behavior in question, this is reinforced, positive consequences of the self-care behavior are stressed, and/or ideas are provided on how to keep up motivation. If a user finds the self-care behavior in question “a little important”, understanding for the users´ ambivalence is uttered, and the importance the user attaches to the self-care behavior – little as it might be – is stressed and reinforced. Finally, if a user rates the self-care behavior as not important, the autonomy expressed in this answer is respected in order not to elicit resistance.

#### Control condition

**Figure 3:**
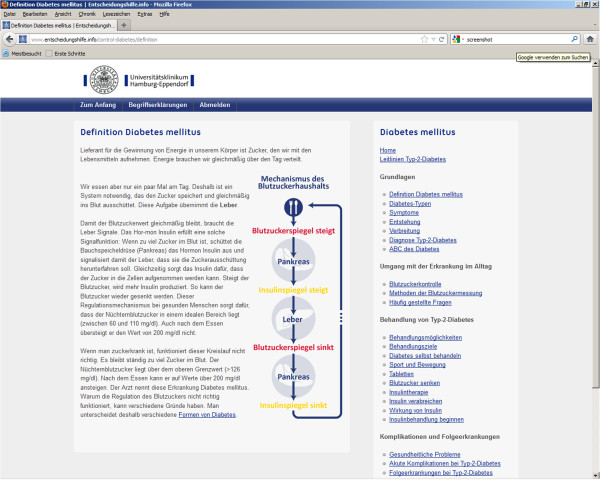
Control window.

On the standard website, the content is not tailored and is not presented in a dialogue format. In contrast to the tailored, interactive version, it is not tunnelled; there is no guidance through the content. On the right of each page a content tree displays a menu of all content sections that the participant can click on to get to the content of interest (see Figure 3).

### Potential risk for participants

Contraindications or side effects of IHCAs are not known.

### Intervention development and trial design

The development process was user-oriented, evidence-based and peer reviewed. In order to find out which topics are relevant to patients with type 2 diabetes, we performed a needs assessment with two steps: First, semi-structured interviews with seven physicians (all internists, 1 working as a general practitioner, 2 specialized in diabetology) and ten patients with type 2 diabetes were conducted. In the second step, a self-assessment questionnaire was developed based on the main results of the interviews, and it was administered to a new and larger patient sample (N=178). The needs assessment will be described in more detail elsewhere. In order to ensure that information is evidence-based treatment guidelines were used as primary sources. Based on review articles [32,33], expert advice and up-to-dateness, the British [34] and the American [26] guidelines were chosen. Throughout the development, the content was revised by an interdisciplinary advisory committee in an iterative process. The development will be described in more detail elsewhere.

### Outcome assessment

The primary outcomes are diabetes knowledge (assessed immediately after the first visit) and patient empowerment (assessed at three month follow-up). Diabetes knowledge is assessed with 16 multiple-choice questions we developed according to the IHCA´s content. Patient empowerment is measured with the Health Education Impact Questionnaire (HeiQ) [35,36]. The HeiQ includes 42 items and eight dimensions: Positive and Active Engagement in Life, Health Directed Behavior, Skill and Technique Acquisition, Constructive Attitudes and Approaches, Self-Monitoring and Insight, Health Service Navigation, Social Integration and Support, and Emotional Wellbeing. Schuler and colleagues [37] translated the questionnaire into German and evaluated its psychometric properties (Raykov’s Composite Reliability Coefficient, factorial and concurrent validity). They were able to replicate the structure of the eight scales and found the questionnaire to be a reliable and valid measure. We removed Social Integration and Support from our testing battery since we did not expect an effect of our IHCA on that dimension.

Secondary outcomes are decisional conflict and preparation for decision making. Decisional conflict is assessed with the Decisional Conflict Scale (DCS) by O´Connor [38]. This questionnaire measures personal perceptions of uncertainty in choosing options, modifiable factors contributing to uncertainty such as feeling uninformed, unclear about personal values and unsupported in decision making, and effective decision making such as feeling the choice is informed, values-based, likely to be implemented, and expressing satisfaction with the choice. Reliability is good with a Cronbach´s α between 0.78 and 0.92 [38]. Discriminant validity is acceptable.

Preparation for decision making is measured with the Preparation for Decision Making Scale (PDMS) [39]. This 11 item scale assesses a patient´s or participant´s perception of how useful a decision aid or decision support intervention was in preparing him or her to communicate with his or her practitioner in making a health decision. Reliability is very good ranging from α=.92 to α=.94. Both questionnaires are offered only to those participants who have indicated that they are facing a health decision concerning their type 2 diabetes. In order to avoid missing data, all questionnaires include validation checks that alert participants when their answers are implausible or items are skipped.

### Statistical analyses

T-tests for independent samples will be performed to test the hypotheses. Due to randomization and the supposed structural equality of the groups we do not expect confounding factors. If we detect baseline disparities between the control and intervention group they will be included in an analysis of covariance (ANCOVA) as confounding variables. Following the intention-to-treat approach we will include all randomized participants in the analyses in order to avoid biases such as non-random attrition of participants. Additionally we will perform a sensitivity analysis following the per-protocol approach including only participants that have filled in all the questionnaires. For all analyses α ≤ 0.05 will be the critical value for statistical significance. We expect only small sample sizes with respect to the exploratory research questions because only a fraction of the participants will be facing a health decision and will therefore be asked to fill in the DCS and PDMS. For all parameters 95% confidence intervals will be defined so we will be able to appraise the exactitude of testing.

### Power calculation

On the basis of the Cochrane review by Murray et al. [11] we expect a small effect on the primary outcomes (Cohen’s d=0.2). To detect a small effect with an α of 0.05 and a power of 0.80 (one-tailed t-test), a sample size of N=310 (155 per group) is required. Expecting a rate of dropout 20% between registration and follow-up (3 months), we aim at including a sample of N=414 at baseline.

## Discussion

In an on-going RCT, we are testing a web-based, tailored, dialogue-based information system that contains information on type 2 diabetes and its sequelae, health behaviour, and treatment options, against a standard website providing identical information without dialogue structure, tailoring or interactive elements. Both websites were thoroughly developed based on a needs assessment and two evidence-based guidelines, and reviewed by an interdisciplinary advisory committee. The primary outcomes of the trial are diabetes knowledge and patient empowerment. Secondary outcomes are decisional conflict, preparation for decision making, and website usage. The present study is the first trial on a German language IHCA on type 2 diabetes.

There are some limitations to the work presented. The most obvious limitation is that only people with Internet access can be included in the study. 73% of the German general population use the Internet [40], but of the population over 50 years of age, 47% are online. Since the prevalence of type 2 diabetes increases strongly with age [41] we run the risk of excluding a part of our target group. This is a limitation both with respect to implementation and reach, and as a source of selection bias.

There are some disadvantages of online questionnaires, namely the relatively high nonresponse rates and concerns regarding data quality [42,43]. With regard to the quality of the data obtained online, there are indications that the psychometric properties are equivalent with data obtained from paper pencil questionnaires or even better [44,45]. Quality can be improved by validation checks that alert participants when their answers are implausible or items are skipped [44]. Furthermore, online assessments seem to be less prone to social desirability [46]. With respect to non-responders we try to reduce attrition by keeping the questionnaires as short as possible, making the intervention itself attractive, and offering an incentive for answering all questionnaires. Another limitation concerning our measurements is that only some of them are standardized (DCS, PDMS, BIT) while others are adapted (attitudes toward self-care) or developed (diabetes knowledge) for our purposes. None of the measurements have been adapted for online use which puts their comparability to results obtained from paper pencil tests into question [47].

## Abbreviations

SDM: Shared decision-making; IHCA: Interactive Health Communication Application; RCT: Randomized controlled trial; PDMS: Preparation for Decision Making Scale; DCS: Decisional Conflict Scale; HeiQ: Health Education Impact Questionnaire; ADA: American Diabetes Association; oGTT: Oral Glucose Tolerance Test; ANCOVA: Analysis of covariance.

## Competing interests

The authors declare that they have no competing interests.

## Authors’ contributions

NW participated in the conception and design of the study and drafted the manuscript. MH participated in the conception and design of the study and revised the manuscript. JD participated in the conception and design of the study and revised the manuscript. All authors read and approved the final manuscript.

## Authors’ information

NW is a certified psychotherapist and a research associate at the Department of Medical Psychology. MH is a medical doctor, a certified psychotherapist and head of the Department of Medical Psychology. JD is a certified psychotherapist and a research associate at the Department of Medical Psychology.

## Pre-publication history

The pre-publication history for this paper can be accessed here:

http://www.biomedcentral.com/1472-6947/13/24/prepub
